# Depression and Hypomagnesemia as Independent and Synergistic Predictors of Cognitive Impairment in Older Adults Post-COVID-19: A Prospective Cohort Study

**DOI:** 10.3390/medsci13030114

**Published:** 2025-08-06

**Authors:** José Guzmán-Esquivel, Brando S. Becerra-Galindo, Gustavo A. Hernández-Fuentes, Marco A. Ramos-Rojas, Osiris G. Delgado-Enciso, Hannah P. Guzmán-Solórzano, Janet Diaz-Martinez, Verónica M. Guzmán-Sandoval, Carmen A. Sanchez-Ramirez, Valery Melnikov, Héctor Ochoa-Diaz-Lopez, Daniel Montes-Galindo, Fabian Rojas-Larios, Iván Delgado-Enciso

**Affiliations:** 1 Clinical Epidemiology Research Unit, Mexican Institute of Social Security, Villa de Alvarez, Colima 29883, Mexico; jose.esquivel@imss.gob.mx (J.G.-E.); hannah_gz@hotmail.com (H.P.G.-S.); 2 Department of Geriatrics, Mexican Institute of Social Security (IMSS), General Hospital of Zone No. 1, Villa de Alvarez, Colima 28984, Mexico; brando_becerra@ucol.mx (B.S.B.-G.); marcoz74@hotmail.com (M.A.R.-R.); 3 Department of Molecular Medicine, School of Medicine, University of Colima, Colima 28040, Mexico; gahfuentes@gmail.com (G.A.H.-F.); 1933osiris@gmail.com (O.G.D.-E.); carmen_sanchez@ucol.mx (C.A.S.-R.); valery.melnikoff@gmail.com (V.M.); frojas@ucol.mx (F.R.-L.); 4 Faculty of Chemical Sciences, University of Colima, Coquimatlan 28400, Mexico; da.montesg@gmail.com; 5 State Cancerology Institute of Colima, Health Services of the Mexican Social Security Institute for Welfare (IMSS-BIENESTAR), Colima 28085, Mexico; 6 Research Center in Minority Institutions, Florida International University (FIU-RCMI), Miami, FL 33199, USA; jdimarti@fiu.edu; 7 Department of Dietetics & Nutrition, Robert Stempel College of Public Health & Social Work, Florida International University (FIU-RCMI), Miami, FL 33199, USA; 8 School of Psychology, University of Colima, Colima 28040, Mexico; gus_vero@ucol.mx; 9 Department of Health, El Colegio de La Frontera Sur, San Cristobal de Las Casas 29290, Mexico; hochoa@ecosur.mx

**Keywords:** COVID-19, cognitive impairment, older adults, depression, hypomagnesemia, risk factors

## Abstract

**Background/Objectives:** Cognitive impairment in older adults has emerged as a growing public health concern, particularly in relation to COVID-19 infection and its associated neuropsychiatric symptoms. The identification of modifiable risk factors may contribute to the development of targeted preventive strategies. This study aimed to assess predictors of cognitive impairment in older adults with and without recent SARS-CoV-2 infection. **Methods:** A prospective cohort study was conducted from June 2023 to March 2024 at a tertiary hospital in western Mexico. Adults aged 65 years or older with confirmed SARS-CoV-2 infection within the previous six months, along with uninfected controls, were enrolled. Cognitive function (Mini-Mental State Examination), depression (PHQ-9), anxiety (Geriatric Anxiety Inventory), insomnia (Insomnia Severity Index), functional status (Katz Index and Lawton–Brody Scale), and laboratory markers were evaluated at baseline, three months, and six months. The primary outcome was cognitive impairment at six months. Independent predictors were identified using a multivariable generalized linear mixed-effects model. **Results:** Among the 111 participants, 20 (18.8%) developed cognitive impairment within six months. Low serum magnesium (adjusted risk ratio [aRR] 2.73; 95% CI 1.04–7.17; *p* = 0.041) and depression (aRR 5.57; 95% CI 1.88–16.48; *p* = 0.002) were independently associated with a higher risk. A significant synergistic among COVID-19, depression, and hypomagnesemia was observed (RR 44.30; 95% CI 9.52–206.21; *p* < 0.001), corresponding to the group with simultaneous presence of all three factors compared to the group with none. **Conclusions:** Depression and hypomagnesemia appear to be independent predictors of cognitive impairment in older adults with recent COVID-19 infection. These findings suggest potential targets for prevention and support the implementation of routine neuropsychiatric and biochemical assessments in this population.

## 1. Introduction

Given the increasing life expectancy and the associated burden of age-related morbidities, cognitive impairment in older adults is a growing concern globally. It is estimated that mild cognitive impairment occurs in approximately 3–22% of adults over 65 years of age, and a substantial proportion progress to dementia within five years [1]. The etiology of cognitive impairment is multifactorial, involving neurodegenerative processes, vascular changes, metabolic dysregulation, psychiatric comorbidities, and systemic inflammation [2]. Acute infections, particularly COVID-19, have received increased attention for their possible ability to hasten or reveal cognitive impairment in older adults who were previously healthy [3].

Since SARS-CoV-2 first surfaced, a growing amount of data have demonstrated the virus’s ability to impact the central nervous system (CNS) through endothelial dysfunction, systemic immune activation, direct neuroinvasion, or chronic neuroinflammation [3,4]. Post-acute sequelae of COVID-19, also known as long COVID, encompass a range of persistent symptoms, including fatigue, sleep disorders, depression, anxiety, and so-called “brain fog” (a colloquial term often used by patients to describe subjective cognitive dysfunction) [5,6]. While much of this evidence comes from retrospective cohorts or transversal studies, prospective data evaluating the trajectory and risk factors for cognitive impairment in older COVID-19 survivors remain scarce, especially from a multifactorial dimension.

Neuropsychiatric symptoms such as depression, anxiety, and insomnia are well-established risk factors for cognitive impairment in the elderly [7]. These conditions have overlapping pathophysiological mechanisms with cognitive disorders. Moreover, the recent literature suggests that the interplay between psychiatric symptoms and biological factors—such as metabolic alterations or electrolyte imbalances—could significantly influence long-term cognitive outcomes [4,7,8].

Electrolyte disturbances, particularly involving magnesium, calcium, and sodium, are prevalent among older adults due to age-related physiological changes, dietary insufficiencies, polypharmacy, and comorbid chronic diseases [9]. Magnesium, in particular, has garnered attention due to its critical role in neuromuscular transmission, synaptic plasticity, and NMDA receptor regulation, which are mechanisms that are central to cognitive processing and memory formation. Low intakes of Mg and Ca and low serum Mg levels are beginning to be associated with an increased incidence of dementia in some studies [10,11,12]. The potential synergistic effect of magnesium deficiency and COVID-19-related neuroinflammatory processes on long-term cognition has not been systematically examined.

To date, few prospective studies have stratified older adults by COVID-19 status and followed them longitudinally to assess cognitive outcomes. Furthermore, most existing studies lack baseline cognitive assessments, do not control neuropsychiatric symptoms, or fail to incorporate biological markers, such as serum electrolytes, that may provide insight into modifiable risk pathways. Given the complexity and multifactorial nature of cognitive impairment, there is a pressing need for research that integrates clinical, psychological, and biochemical domains to identify high-risk individuals and potential targets for intervention.

In this context, a prospective cohort study was conducted among community-dwelling older adults in western Mexico, all functionally independent and cognitively intact at baseline. The primary objective was to compare individuals with recent COVID-19 infection to matched controls with no history of SARS-CoV-2 exposure, to assess the prevalence of cognitive impairment over a six-month follow-up period. Additionally, the study aimed to identify clinical and laboratory indicators associated with cognitive impairment, with a focus on depression, anxiety, insomnia, and serum electrolyte abnormalities, particularly calcium and magnesium levels.

It was hypothesized that COVID-19 infection would be associated with an increased risk of cognitive impairment at three and six months, and that this association could be influenced by the presence of neuropsychiatric symptoms and biochemical imbalances. Special attention was given to exploring a potential interaction between depression, low magnesium levels, and COVID-19 exposure as a combined risk profile for cognitive impairment. A multivariable mixed-effects modeling approach was employed to account for repeated measures and to identify independent predictors after adjustment for relevant covariates.

By leveraging prospective data and a multidimensional assessment protocol, including neuropsychological testing, validated psychiatric screening tools, and laboratory biomarkers, this study sought to provide a comprehensive characterization of cognitive trajectories in older adults after COVID-19 infection. The findings may offer valuable insights for post-infection monitoring, preventive strategies, and the development of targeted interventions aimed at preserving cognitive health in vulnerable elderly populations.

## 2. Materials and Methods

### 2.1. Study Design and Participants

This was a prospective cohort study conducted between June 2023 and March 2024 at a tertiary care referral hospital in western Mexico (Hospital General de Zona 1, IMSS-Colima). The study included community-dwelling older adults aged 65 years or older, of both sexes, and independence according to the Katz Index of Independence in Activities of Daily Living. All participants had normal cognitive status at baseline and no history of SARS-CoV-2 infection in the control group.

Participants were stratified into two groups based on COVID-19 status: a case group consisting of individuals with laboratory-confirmed SARS-CoV-2 infection (via RT-PCR or rapid antigen test) within six months prior to enrollment, and a control group with no history or clinical evidence of COVID-19 infection before recruitment. Exclusion criteria were pre-existing cognitive impairment or any neuropsychiatric disorder; use of neuropsychiatric or neurological medications (recent or chronic); history of stroke in the past 6 months; chronic kidney disease or serum creatinine > 3.0 mg/dL; recent surgery or trauma (within the previous two months); active malignancy; liver cirrhosis; chronic inflammatory conditions; and chronic obstructive pulmonary disease. COVID-19 infection during follow-up was an exclusion criterion.

The study was conducted in accordance with the principles of the Declaration of Helsinki. Approval was obtained from the institutional ethics and research committee (approval code: R-2023-601-034). All participants provided informed consent, and their confidentiality and anonymity were strictly preserved throughout the study.

### 2.2. Procedures and Assessments

All participants underwent comprehensive evaluation at three time points: baseline, 3 months, and 6 months. The primary outcome was the development of cognitive impairment at 3 and/or 6 months, defined as an MMSE score ≤ 24 in participants who had normal cognitive function at baseline. At each visit, the following assessments were conducted:

(1) Cognitive assessment: The Mini-Mental State Examination (MMSE) was used to assess global cognitive function [13]. A score of ≤24 was defined as cognitive impairment. The cutoff point of ≤24 on the Mini-Mental State Examination (MMSE) was chosen to define cognitive impairment based on validated norms for populations with low educational attainment, similar to our cohort, which had an average of approximately 4 years of formal education [14]. Using a higher cutoff such as <27, common in populations with higher education, could lead to overestimation of cognitive impairment in this context. This cutoff aims to balance sensitivity and specificity for detecting clinically relevant impairment in this population [15,16].

(2) Neuropsychiatric symptoms: Depression was evaluated using the PHQ-9 scale. Scores >10 indicated moderate-to-severe depression [17]. Anxiety was assessed with the Geriatric Anxiety Inventory (GAI), considered positive with scores equal to or greater than nine [18]; and insomnia with the Insomnia Severity Index (ISI) [18,19], categorizing its presence with a score of eight or more (from mild to severe insomnia).

(3) Laboratory tests: Blood samples were analyzed for creatinine, urea, hemoglobin, and serum electrolytes, including sodium (Na), potassium (K), total serum calcium (Ca), and magnesium (Mg). Electrolyte disturbances were defined as follows: Na < 135 mEq/L, K < 3.5 mEq/L, Ca < 8.5 mg/dL, Mg < 1.7 mg/dL, urea > 43 mg/dL, and creatinine > 1.18 mg/dL.

### 2.3. Statistical Analysis

Continuous variables were described using means and standard deviations and compared using Student’s *t*-test for normally distributed data, which was assessed using the Kolmogorov–Smirnov test. Categorical variables were compared using Fisher’s exact tests. To evaluate predictors of cognitive impairment, a multivariable generalized linear mixed-effects model with a binary logistic link was applied. The timing of assessment (baseline, 3 months, and 6 months) was included as a random effect to account for within-subject variability over time. All variables with *p* < 0.05 in bivariable analysis were included in the multivariable model. The results are reported as relative risks (RRs) or adjusted relative risks (aRRs) with 95% confidence intervals (CI) and corresponding *p*-values. To explore the interaction between COVID-19, depression, and low magnesium levels, a three-way interaction model was conducted using a multivariable mixed-effects framework. COVID-19 status, presence of depression, low magnesium, and their interaction terms were included as fixed effects. Time points was modeled as a random effect. This model allowed the assessment of whether the combination of these factors had a synergistic (multiplicative) effect on cognitive impairment, beyond their individual contributions [20]. All statistical analyzes were conducted using SPSS version 26.0 (IBM Corp., Armonk, NY, USA). A *p*-value < 0.05 was considered statistically significant [21].

## 3. Results

### 3.1. Clinical Characteristics Associated with Cognitive Impairment at Six Months

**Figure 1:**
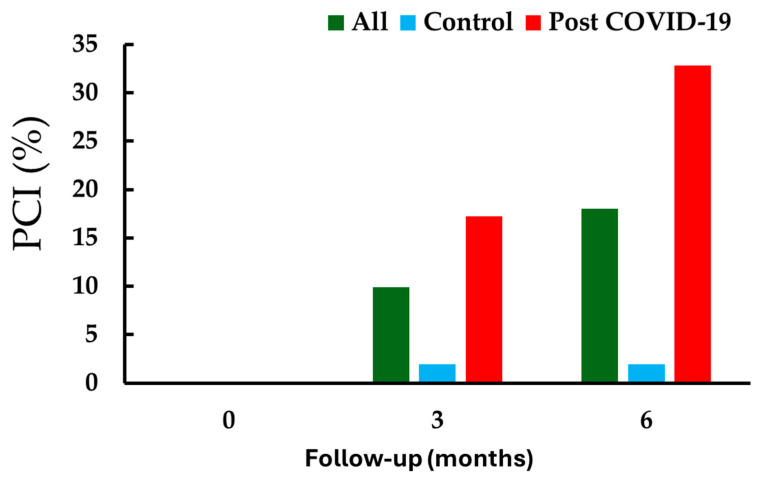
Patients with cognitive impairment (PCI, %) at baseline, 3 months, and 6 months of follow-up, stratified by history of COVID-19 infection. At baseline, no participant was presented with cognitive impairment. At 3 and 6 months, cognitive impairment was observed in 9.9% and 18.8% of all participants, respectively (within-group analysis, before vs. after, *p* < 0.001). Among controls, only 1.9% exhibited cognitive impairment at both 3 and 6 months (within-group analysis, *p* = 0.442). In contrast, among patients with a history of COVID-19, cognitive impairment was present at 17.2% at 3 months and 32.8% at 6 months (within-group analysis, *p* < 0.001). Between-group comparisons showed a significant difference between COVID-19 and control groups at both points (3 months: *p* = 0.009; 6 months: *p* < 0.001).

In this cohort of 111 patients, 20 (18.8%) developed cognitive impairment by the six-month follow-up, with none showing impairment at baseline (see Figure 1); no subjects were excluded due to COVID-19 infection during follow-up. When comparing those with and without cognitive impairment at the six-month follow-up, there were no notable differences in sex distribution, age, oxygen saturation, or years of education.

Age (mean ± SD: 73.7 ± 5.8 vs. 75.6 ± 5.6 years; *p* = 0.197) and sex distribution (male: 56.0% vs. 55.0%; *p* = 0.999) did not differ significantly between groups. Years of education and oxygen saturation levels also did not differ substantially. Patients with cognitive impairment had lower rates of hypertension (25.0% vs. 57.1%; *p* = 0.013). Creatinine and anemia prevalence were comparable between groups, despite urea levels being generally lower in the cognitive impairment group (45.0% vs. 69.2%; *p* = 0.068).

Significant relationships were found between electrolyte abnormalities and cognitive impairment: low magnesium (40.0% vs. 11.0%; *p* = 0.004) and low calcium (60.0% vs. 29.7%; *p* = 0.018) were more common in this group of patients. Low potassium and low sodium did not significantly affect the results. Those with cognitive impairment were substantially more likely to experience neuropsychiatric symptoms, such as anxiety (40.0% vs. 6.6%; *p* < 0.001), depression (60.0% vs. 14.3%; *p* < 0.001), and insomnia (35.0% vs. 5.5%; *p* = 0.001).

Lastly, the cognitive impairment group had a substantially greater incidence of a history of COVID-19 infection (95.0% vs. 42.9%; *p* < 0.001). According to these results, cognitive impairment at six months in this population may be related to low calcium and magnesium levels, depression, anxiety, insomnia, and a history of COVID-19 infection. The temporal relationship between these factors is reinforced by the absence of cognitive impairment at baseline. It is important to note that the magnesium-to-calcium ratio—previously identified as a predictor of mortality in the acute phase of COVID-19 [22]—did not differ between patients with and without cognitive impairment at the six-month follow-up. The main clinical characteristics are present in Table 1.

### 3.2. Predictors of Cognitive Impairment at Six Months: Multivariable Analysis Results

The relative risk (RR) of cognitive impairment at six months was determined using a multivariable generalized linear mixed model with a binary logistic regression link (Table 2). The follow-up period timepoints (baseline, 3 months, and 6 months) were included as a single random effect to account for within-subject variability over time. Variables that showed statistical significance in the bivariable analysis were subsequently included in the multivariable model.

In the bivariable analysis, hypocalcemia (RR 3.33; 95% CI 1.28–8.67; *p* = 0.014), hypomagnesemia (RR 3.79; 95% CI 1.27–11.31; *p* = 0.017), depression (RR 11.58; 95% CI 5.29–25.37; *p* < 0.001), anxiety (RR 9.47; 95% CI 3.91–22.92; *p* < 0.001), insomnia (RR 11.43; 95% CI 4.38–29.83; *p* < 0.001), and post-COVID-19 status (RR 15.21; 95% CI 3.21–72.08; *p* = 0.001) were all significantly associated with an increased risk of cognitive impairment.

After adjustment in the multivariable model, only depression (adjusted RR [aRR] 5.57; 95% CI 1.88–16.48; *p* = 0.002) and hypomagnesemia (aRR 2.73; 95% CI 1.04–7.17; *p* = 0.041) remained independently associated with a higher risk of cognitive impairment at six months. Other variables that were significant in the bivariable analysis—such as anxiety, insomnia, hypocalcemia, and post-COVID-19 status—lost statistical significance after adjustment.

In both bivariable and multivariable analyses, no statistically significant associations were found for sex, age, years of education, type 2 diabetes mellitus, hypertension, uremia, creatinine levels, anemia, electrolyte disturbances, such as hyponatremia or hypokalemia, or alcohol and tobacco use. These findings highlight depression and magnesium status as independent predictors of cognitive impairment, underscoring potential targets for intervention and follow-up in this population.

### 3.3. Interaction Effects of COVID-19, Depression, and Low Magnesium on Cognitive Impairment Risk at Six Months

The combined effects of depression, low magnesium levels, and COVID-19 infection on the risk of cognitive impairment at six months were evaluated using a multivariable mixed-effects model. These three factors, along with their three-way interaction, have been included as fixed effects in this model. The follow-up period timepoints (baseline, three months, and six months) were modeled as a random effect (Table 3).

The findings showed that the three variables had a strong synergistic effect. When compared to those without any of these risk factors, patients with depression, low magnesium, and concurrent COVID-19 had the highest relative risk of cognitive impairment (RR 44.30; 95% CI 9.52–206.21; *p* < 0.001). Normal magnesium levels but COVID-19 and depression were also linked to a significantly higher risk (RR 7.47; 95% CI 2.74–20.39; *p* < 0.001).

In this cohort, COVID-19 by itself did not substantially raise risk in the absence of depression or low magnesium in this cohort (RR 1.48; 95% CI 0.58–3.80; *p* = 0.415). In the absence of COVID-19, neither depression nor low magnesium by themselves were linked to a significant increase in risk. These results underline the significance of thorough clinical evaluation and possible targeted interventions in impacted patients by pointing to a multiplicative interaction between COVID-19, depression, and magnesium deficiency in causing cognitive impairment.

## 4. Discussion

With increasing evidence that SARS-CoV-2 infection can cause or worsen neurocognitive impairments, COVID-19 has become an important factor in the development of cognitive impairment in older adults [23]. However, the current prospective study shows that modifiable factors like depression and low serum magnesium levels play important roles in determining cognitive outcomes in this population, even beyond the direct effects of the virus. Our research indicates a multifactorial and possibly targetable pathway fundamental to post-COVID cognitive impairment, as these factors not only independently increase the risk of cognitive impairment but also work in concert with COVID-19 exposure. By moving the emphasis from viral infection alone to a more comprehensive biopsychosocial model that takes into account psychiatric and biochemical determinants, this nuanced understanding advances the field.

Our results are consistent with earlier studies showing that COVID-19 negatively affects older adults’ cognitive function. For example, a recent observational study found that older COVID-19 patients had higher rates of institutionalization, accelerated reductions in Mini-Mental State Examination (MMSE) scores, and a threefold increase in cognitive impairment when compared to uninfected controls [24]. In a similar vein, cross-sectional studies have connected memory, attention, and executive function deficits to systemic immune activation and chronic neuroinflammation after COVID-19 [25,26]. However, causal inference has been limited in many previous studies due to retrospective designs or the absence of baseline cognitive assessments. With six months of repeated cognitive and clinical assessments, our prospective design offers solid temporal evidence that COVID-19 is linked to incident cognitive impairment in older adults who were previously cognitively intact and living in the community.

Importantly, in line with established research on neuropsychiatric symptoms as risk factors for dementia and cognitive impairment in the elderly [27], our study emphasizes the significant role of depression as an independent predictor of cognitive impairment following COVID-19. Through processes like reduced neurotrophic support, hypothalamic–pituitary–adrenal axis dysregulation, and chronic neuroinflammation, depression may exacerbate cognitive impairment [28,29,30]. In order to potentially lessen cognitive sequelae, our cohort’s high relative risk for depression emphasizes the necessity of routinely screening for and treating depressive symptoms in older COVID-19 survivors.

The cause of depression in post-COVID-19 patients is likely multifactorial, involving both biological and psychological factors. Biological mechanisms may include neuroinflammation, dysregulation of the hypothalamic–pituitary–adrenal (HPA) axis, and direct viral effects on the central nervous system. Psychological contributors may arise from social isolation, grief, economic stress, and lifestyle disruptions caused by the pandemic. Current evidence highlights the complexity of these interactions but remains inconclusive regarding their relative contributions. Therefore, longer-term follow-up studies are essential to disentangle the biological and psychosocial determinants of depression in COVID-19 survivors and to understand their impact on cognitive outcomes. This aligns with recent global analyses of the increased burden of depressive and anxiety disorders during the pandemic [31].

The recognition that low serum magnesium is a separate risk factor for cognitive impairment is equally significant. For cognitive processing and memory formation, magnesium is crucial for neuronal excitability, synaptic plasticity, and N-methyl D-aspartate (NMDA) receptors [32,33]. NMDA receptors play an important role in the process of learning and memory formation NMDA receptor activity regulation [32]. Due to dietary deficiencies, polypharmacy, and age-related physiological changes, magnesium deficiency is common in older adults [9]. Our findings are among the first to prospectively link magnesium deficiency with cognitive impairment in the context of COVID-19, despite previous research suggesting associations between magnesium status and dementia risk. Magnesium’s immunomodulatory properties, which may reduce neuroinflammation brought on by SARS-CoV-2 infection, lend biological plausibility to the theory [33,34].

It has been postulated that magnesium deficiency can exacerbate the inflammatory response in COVID-19 patients [34]. This suggests that taking magnesium supplements could be an inexpensive, easily accessible way to help at-risk individuals maintain their cognitive function. It has previously been postulated that Magnesium may be an effective therapy for Alzheimer’s disease [35]; however, this is a hypothesis that should be analyzed in future studies.

In line with these observations, previous studies have explored the association between hypomagnesemia, COVID-19, the respiratory tract, and lung disease [36], reporting that magnesium deficiency may play a critical role in the severity of respiratory infections, including COVID-19. Moreover, the recent literature has reviewed the potential role of zinc and/or magnesium in enhancing the effectiveness of pharmacological therapies or mitigating adverse effects of anti-COVID-19 drugs. In our bivariable analysis, both low magnesium and low calcium levels were significantly associated with cognitive impairment at six months. This highlights the importance of monitoring and potentially supplementing these ions during and after COVID-19 infection. While magnesium has received greater attention due to its role in inflammation and neuroprotection, calcium also plays a crucial role in neurotransmission, long-term potentiation, and the synthesis of neurotransmitters involved in memory and learning [37]. The importance of initiating oral magnesium trials in COVID-19 patients is also emphasized [38], further reinforcing the clinical relevance of these trace elements. It is important to note that the pathophysiology of post-COVID cognitive impairment appears to depend primarily on suboptimal absolute serum levels of both magnesium and calcium, rather than on their ratio. The brain is vulnerable to deficiencies in either ion, even if the magnesium-to-calcium ratio remains within a normal range. This explains why both ions differed significantly between patients with and without cognitive impairment, while their ratio did not distinguish those who would develop long-term cognitive decline.

A particularly strong synergistic relationship was observed between depression, hypomagnesemia, and COVID-19 infection. The risk of cognitive impairment was over 40 times higher in individuals presenting with all three factors compared to those without, suggesting a multiplicative rather than additive effect [38]. This interaction likely reflects the combined impact of virus-induced neuroinflammation, neurobiological alterations associated with mood disorders, and biochemical vulnerability linked to magnesium deficiency, which together increase the risk of cognitive impairment. Notably, COVID-19 infection alone—without concurrent depression or hypomagnesemia—did not significantly elevate the risk of cognitive impairment. This finding is in line with recent studies demonstrating that the presence of psychiatric and metabolic comorbidities, rather than the infection itself, is more predictive of post-COVID neurocognitive decline [39,40]. These results emphasize the need for comprehensive biopsychosocial assessments to identify individuals truly at heightened risk and to guide targeted interventions [40].

Additionally, a study analyzing the peripheral blood of patients with severe COVID-19 [41] found significantly elevated levels of ferritin compared to patients with non-severe disease. These findings suggest that serum ferritin may serve as a biomarker of disease severity and systemic inflammation in COVID-19. Although our study included laboratory markers such as serum calcium and other electrolytes, ferritin levels were not measured. Furthermore, antioxidant capacity and more specific inflammatory biomarkers, such as cytokine panels, were not assessed, and neuroimaging data (e.g., MRI) were unavailable due to resource limitations. Routine tests like complete blood count (CBC) and erythrocyte sedimentation rate (ESR) were performed but are less specific. Therefore, incorporating ferritin and a broader panel of inflammatory and oxidative stress markers, along with neuroimaging, in future studies could represent a valuable strategy to better understand and support the association between systemic inflammation and long-term neuropsychiatric and cognitive outcomes in older adults recovering from COVID-19.

Numerous clinical and public health implications result from our study. To identify people at risk for cognitive impairment early on, it first supports the inclusion of cognitive and psychiatric screening in routine post-COVID care for older adults. Second, measuring serum magnesium levels could offer a useful, adjustable biomarker to direct focused treatments. Third, to lessen the long-term cognitive effects of COVID-19, multidisciplinary care that considers both nutritional status and mental health may be crucial. There is an immediate need for scalable strategies that incorporate these components, given the anticipated rise in older COVID-19 survivors worldwide.

Our study’s prospective design, well-characterized cohort with baseline cognitive intactness, and extensive multidimensional assessments that include cognitive testing, psychiatric evaluation, and laboratory biomarkers are among its strong points. Strong analysis of longitudinal data and the relationships between several risk factors were made possible by the application of mixed-effects modeling. Limitations must be recognized, though. Generalizability may be limited by the small sample size and the fact that it came from a single center in western Mexico. The MMSE, which may not pick up on subtle domain-specific deficiencies, was the main tool used for cognitive assessment. It is necessary to conduct more research using comprehensive neuropsychological tests and neuroimaging. Additionally, the severity of COVID-19 infection was not stratified, limiting conclusions about its differential impact on cognitive outcomes. Moreover, while depression was thoroughly assessed, other neuropsychiatric conditions were not comprehensively evaluated. Finally, the absence of neuroimaging data, vitamin D levels of the patients, pharmacological registrum, and biomarkers of neuroinflammation or neurodegeneration, as well as the relatively short follow-up period of six months, limits insights into the underlying mechanisms and the long-term trajectory of cognitive impairment in this population. Future studies should address these gaps by including larger, more diverse cohorts, repeated biomarker assessments, detailed neuropsychiatric evaluations, and extended longitudinal follow-up with neuroimaging support. In addition, studies should also consider other trace elements such as phosphorus, which, like calcium and magnesium, may play a significant role in the metabolic imbalances observed in COVID-19 patients and could be associated with neurological or psychiatric manifestations [42].

## 5. Conclusions

In conclusion, the study shows that depression and low serum magnesium levels are important modulatory determinants that significantly influence cognitive outcomes in this context, even though COVID-19 is a relevant and significant factor in the development of cognitive impairment among older adults. In order to maintain cognitive health in vulnerable older populations, these findings support a multifactorial, integrative model of post-COVID cognitive impairment and identify specific areas that warrant clinical intervention.

## Figures and Tables

**Table 1:** Comparison of the main clinical characteristics according to the presence of cognitive impairment at six-month follow-up.

	Number *		Cognitive Impairment		
Clinical Characteristic		All *n* = 111	No *n* = 91	Yes *n* = 20	*p*
Male	62	55.9%	56.0%	55.0%	0.999
Age, mean ± SD (years)	--	74.03 ± 5.80	73.69 ± 5.82	75.55 ± 5.60	0.197
Oxygen saturation, mean ± SD (%)	--	92.77 ± 2.07	92.78 ± 1.96	92.70 ± 2.53	0.876
Years of education, mean ± SD	--	4.05 ± 3.18	4.29 ± 3.41	3.00 ± 1.48	0.103
Diabetes	68	61.3%	61.5%	60.0%	0.999
Hypertension	57	51.4%	57.1%	25.0%	0.013
Urea	72	64.9%	69.2%	45.0%	0.068
Creatinine	83	74.8%	75.8%	70.0%	0.579
Anemia	82	73.9%	74.7%	70.0%	0.779
Hyponatremia (Na)	31	27.9%	26.4%	35.0%	0.424
Hypokalemia (K)	11	9.9%	8.8%	15.0%	0.414
Hypocalcemia (Ca)	39	35.1%	29.7%	60.0%	0.018
Hypomagnesemia (Mg)	18	16.2%	11.0%	40.0%	0.004
Mg–Ca ratio	--	0.22 ± 0.05	0.22 ± 0.05	0.23 ± 0.06	0.628
Tobacco use	25	22.5%	22.0%	25.0%	0.772
Alcohol use	38	34.2%	34.1%	35.0%	0.999
Depression	25	22.5%	14.3%	60.0%	<0.001
Anxiety	14	12.6%	6.6%	40.0%	<0.001
Insomnia	12	10.8%	5.5%	35.0%	0.001
Post COVID-19	58	52.3%	42.9%	95.0%	<0.001

No participant had cognitive impairment at baseline. Cut-off points used: creatinine > 1.18 mg/dL; urea > 43 mg/dL; sodium (Na) < 135 mEq/L; potassium (K) < 3.5 mEq/L; calcium (Ca) < 8.5 mg/dL; and magnesium (Mg) < 1.7 mg/dL. * Number = number of patients with the characteristic.

**Table 2:** Relative risk of cognitive impairment at 6 months follow-up: multivariable generalized linear mixed model with binary logistic regression.

	Bivariable Model				Multivariable Model			
	RR	95% CI		*p*	AdRR	95% CI		*p*
		Lower	Upper			Lower	Upper	
Male	0.848	0.407	1.765	0.658				
Age	1.047	0.963	1.138	0.281				
Years of Education	0.580	0.259	1.301	0.186				
Type 2 Diabetes (DM2)	1.086	0.522	2.261	0.824				
HAS	0.580	0.260	1.291	0.181				
Urea	1.050	0.479	2.303	0.903				
Creatinine	1.639	0.747	3.595	0.217				
Anemia	1.859	0.844	4.094	0.123				
Hyponatremia	1.290	0.466	3.573	0.623				
Hypokalemia	1.078	0.231	5.020	0.924				
Hypocalcemia	3.332	1.280	8.672	0.014	1.438	0.484	4.274	0.512
Hypomagnesemia	3.786	1.268	11.309	0.017	2.733	1.041	7.172	0.041
Low Mg–Ca ratio *	1.006	0.459	2.202	0.711				
Tobacco use	0.926	0.342	2.508	0.961				
Alcohol use	1.290	0.712	2.358	0.879				
Depression	11.578	5.285	25.367	<0.001	5.566	1.880	16.476	0.002
Anxiety	9.466	3.909	22.919	<0.001	2.767	0.704	10.874	0.144
Insomnia	11.429	4.379	29.833	<0.001	1.414	0.301	6.631	0.660
Post COVID-19	15.210	3.210	72.077	0.001	1.867	0.613	5.688	0.271

RR = relative risk; aRR = adjusted relative risk; CI = confidence interval; DM2 = type 2 diabetes mellitus; and HAS = hypertension. To assess the relative risk of cognitive impairment at six months, this study used a multivariable generalized linear mixed-effects model with a logistic regression link. In order to capture individual variability over time, the timing of follow-up assessments (baseline, three months, and six months) was the only random effect taken into account. The multivariable model contained variables that were statistically significant in the first bivariable analysis. The 95% CI, corresponding *p*-values, and adjusted relative risks (aRRs) are included in the results. * Low magnesium-to-calcium ratio (≤0.20) was calculated in accordance with a previous study that reported this value to identify people at high risk of mortality in the acute phase of COVID-19 [22].

**Table 3:** Relative risk of cognitive impairment at 6-month follow-up: mixed-effects model with a three-way interaction between COVID-19, depression, and hypomagnesemia.

Presence of Risk Factor			RR	95% CI		*p*
COVID-19	Depression	Hypomagnesemia		Lower	Upper	
No	No	No	1 (Reference)			
Yes	Yes	Yes	44.303	9.518	206.211	<0.001
Yes	Yes	No	17.316	7.106	42.196	<0.001
Yes	No	Yes	7.473	2.738	20.393	<0.001
Yes	No	No	1.478	0.576	3.795	0.415
No	Yes	No	0.788	0.455	1.366	0.395
No	No	Yes	0.788	0.455	1.366	0.395

A multivariable mixed-effects model was used to estimate the relative risk of cognitive impairment at 6 months of follow-up. The model included COVID-19 status, depression, and low magnesium levels, and their three-way interaction as fixed effects. The only random effect incorporated was the follow-up period timepoints (baseline, 3 months, and 6 months) to account for within-subject variability over time. Relative risks (RRs) with 95% confidence intervals (CIs) and *p*-values correspond to contrasts derived from this model.

## Data Availability

The original contributions presented in the study are included in the article; further inquiries can be directed to the corresponding author.
